# Fruit ripening: dynamics and integrated analysis of carotenoids and anthocyanins

**DOI:** 10.1186/s12870-021-03411-w

**Published:** 2022-01-11

**Authors:** Leepica Kapoor, Andrew J. Simkin, C. George Priya Doss, Ramamoorthy Siva

**Affiliations:** 1 Department of Biotechnology, School of Biosciences and Technology, Vellore Institute of Technology, Vellore, Tamil Nadu 632014 India; 2 School of Biosciences, University of Kent, United Kingdom, Canterbury, CT2 7NJ UK

**Keywords:** Fruit ripening, chlorophyll, Carotenoid, Anthocyanin, Pigment dynamics, Biofortification

## Abstract

**Background:**

Fruits are vital food resources as they are loaded with bioactive compounds varying with different stages of ripening. As the fruit ripens, a dynamic color change is observed from green to yellow to red due to the biosynthesis of pigments like chlorophyll, carotenoids, and anthocyanins. Apart from making the fruit attractive and being a visual indicator of the ripening status, pigments add value to a ripened fruit by making them a source of nutraceuticals and industrial products. As the fruit matures, it undergoes biochemical changes which alter the pigment composition of fruits.

**Results:**

The synthesis, degradation and retention pathways of fruit pigments are mediated by hormonal, genetic, and environmental factors. Manipulation of the underlying regulatory mechanisms during fruit ripening suggests ways to enhance the desired pigments in fruits by biotechnological interventions. Here we report, in-depth insight into the dynamics of a pigment change in ripening and the regulatory mechanisms in action.

**Conclusions:**

This review emphasizes the role of pigments as an asset to a ripened fruit as they augment the nutritive value, antioxidant levels and the net carbon gain of fruits; pigments are a source for fruit biofortification have tremendous industrial value along with being a tool to predict the harvest. This report will be of great utility to the harvesters, traders, consumers, and natural product divisions to extract the leading nutraceutical and industrial potential of preferred pigments biosynthesized at different fruit ripening stages.

## Background

Fruits are important and health-promoting food resources and are a part of our diet as a potential source of nutrients [1]. With the development of research and analytical biotechnological approaches, the bioactive compounds in fruits have been identified and considered for their efficacy as nutraceuticals and industrial products. Nutrients like vitamins and minerals cover 85% of the nutraceutical market, followed by antioxidants and herbal extracts. The bioactive compounds present in fruits are utilized as nutraceuticals, cosmetics (sun screen, antiaging formulation,) and in oenology [2]. The multifold uses of fruits have been attributed to the presence of several bioactive compounds, such as chlorophyll, carotenoids, anthocyanins, betalain, phenols, tannins, flavonoids, glycosides, and many more, which are known for their nutraceutical properties and are capable of replacing pharmaceuticals [3]. Among these, fruit pigments like chlorophyll, carotenoids, anthocyanin and betalain have been recognized world-wide as safer natural colorants for several industries such as food and confectionary, textiles, cosmetics, and pharmaceuticals [4, 5].

Fruit color is an indicator of its stage of maturity, freshness and quality and serves as an important parameter in their classification [6]. As the fruit passes through various stages of growth and maturity, the variations in fruit color are observed due to biosynthesis and degradation of pigments in developing fruits. The accumulation of pigments is largely governed by the various maturation stages, during fruit ripening, which in turn is dependent on biotic and abiotic factors along with species’ genetic makeup. During maturation, fruits undergo several biochemical and physiological changes that alter its bioactive composition [7]. The changes in pigments are markers of the development stage and the physiological condition of the fruit which are essential for optimal storage and postharvest management [8]. However, there is lack of data and limited research studying the changes in bioactive compounds during ripening. Also, to date, most of the research on ripening in fruits has been focused on the growth regulatory hormones, but the role of pigments as an asset to ripening has been unexplored. Apart from making the fruits colorful and enhancing its consumer appeal, pigments add value to the ripened fruits by enhancing the net carbon pool, antioxidant status, nutraceutical properties and industrial use. Moreover, pigments could serve as an agro-industrial tool to assess the ripening status and quantifying ripening. Consequently, a strong need was felt to study pigment accumulation in fruits as a platform for future research prospects on biofortification of fruits. Furthermore, the extraction and characterization of pigments in ripened fruits could lead to discovery of novel, unexplored pigments with probable bioactivity and subsequent applications. Keeping this in consideration, here we report pigment dynamics in ripening viz-a-viz regulatory mechanisms involved in pigment biosynthesis, metabolism, stability, and storage along with prospects to enhance desired pigments of nutraceutical and industrial value in fruits.

## Pigments in fruits

Fruit color signifies a genetic trait with ecological and nutritional worth. Plants mainly make use of color for seed dispersion and to attract animals. The other adaptive challenges were entrusted on fruit color by environmental factors and domestication further resulting in diversification. The unprompted mutations occurring repeatedly in pigment biosynthetic pathways lead to variations in fruit color which were often propagated. In the recent decades, the pigmentation pattern was further enriched by introgression breeding coupled with the unravelling of genetic determinants underneath fruit pigments [9].

The emergence of these pigments as a result of fruit ripening has multifold prospects for the growing plant. The chlorophyll content and development of chloroplasts at mature green stage affects the net carbon yield of a ripened fruit [10].

Chlorophyll and carotenoids are the main photosynthetic pigments in plants and play a vital role in enhancing the net carbon yield of the plant [11]. These pigments are embedded in photosystems II and I and are involved in capturing and utilization of light energy by the plant, via the photosynthetic electron transfer and thereby influence the final yield of fruits [11]. It has been reported that green tomato fruit may contribute as much as 10 to 15% of the total fixed carbon of the fruit [12, 13]. Furthermore, the down-regulation of the Calvin-Benson cycle enzyme fructose-1,6-bisphosphatase in green tomato fruit resulted in a 15–20% negative impact on fruit development and yield [14] further demonstrating the importance of chlorophyll to green fruit..

Chlorophyll *a* and *b* are present throughout the photosynthetic machinery whereas carotenoids like β-carotene, violaxanthin, antheraxanthin, zeaxanthin and lutein are mainly present in antenna systems of light harvesting complexes [15, 16]. During periods of high-light stress, plants adopt to the phenomenon of photoinhibition leading to reduced levels of light capture. However, carotenoids inhibit photoinhibition by scavenging the free radicals and undergoing conformational changes (VAZ cycle and Lx Cycle) to protect the plant from the adverse effect of high light [17] and prevent photoinhibition and thereby increase the final carbon pool in the fruits [18]. Also, anthocyanins act as UV filters, and protect the plant from high light at low temperatures, thus reduce photoinhibition and open a window for photosynthesis.

Apart from their crucial role in photosynthesis, pigments like carotenoids and anthocyanins enhance the antioxidant status and therapeutic potential (Table 1) of the fruits along with other compounds like polyphenols, tocopherols, vitamins and catechins [38].

**Table 1:** Pigments as a source of nutraceuticals in fruits

Pigment	Properties	Sources	Health benefits	Reference
**CAROTENOIDS & APOCAROTENOIDS**				
β Carotene	Cyclic carotene, non-polar, high melting point, crystalline, and gives an orange color	Asparagus, apricots, broccoli, carrot, Chinese cabbage, paprika, grapefruit	Precursor of vitamin A, antioxidant, lowers risk of heart diseases, cancers, boosts immune system, and protects from age-related macular degeneration (AMD)	[19] [20]
Lycopene	Non-polar, heat stable, linear structure, and gives a red color	Tomato, watermelon, papaya, carrot, pink grapefruit	Antioxidant, reduces risk of myocardial infarction and high blood pressure, attenuates LDL cholesterol oxidation and risks of prostate, lung, uterine and breast cancer, promotes bone health, delays neurodegeneration	[21] [22] [23]
Lutein and Zeaxanthin	Polar, and gives yellow to red color	Corn, kiwi, orange zucchini, spinach	Protects against (AMD) and cognition	[24] [25]
Bixin	Apocarotenoid, sensitive to light, pH, soluble in organic polar solvents and gives a deep orange color	Annatto (*Bixa orellana*)	Anti-oxidative, anti-cancer, hypoglycemic, antibiotic, anti-inflammatory properties	[26] [27]
Crocin	apocarotenoid, water-soluble and gives an orange color	saffron (*Crocus sativus. L)*	antioxidant, anticancer, antidiabetic, antidepressant, improves cognition and occurrence of autoimmune diseases	[28] [29]
**ANTHOCYANINS**				
Cyanidin, delphinidin, pelargonidin, peonidin, petunidin, malvidin	Water-soluble, vacuolar pigments, sensitive to pH change and can appear as either red, purple, blue or black	Berries, strawberry, eggplant, cherry, black grapes, red cabbage	Potent antioxidant, prevents dyslipidemia and impaired glucose metabolism and possess anti-breast cancer properties,	[30] [31] [32]
**CHLOROPHYLL**				
Chlorophyll *a* Chlorophyll *b*	Green, lipid-soluble, tetrapyrrole derivatives, light harvesting pigments,	Spinach, broccoli, wheat grass, pak choi, rocket salad	Chemo protector, antioxidant properties, detoxifies liver, safeguards against anaemia, and sinusitis, exhibits ergogenic effects	[33] [34]
**BETALAIN**				
Betacyanin Betaxanthin	Water soluble, vacuolar pigments, sensitive to pH, betacyanin give red to violet color, betaxanthin give yellow to orange color	Red beetroot, amaranth, prickly pear, red pitaya	Antioxidant and anti-inflammatory properties, protects against skin and lung cancer, anti-microbial and anti-lipidemic	[35] [36] [37]

Carotenoids like β-carotene, lycopene, zeaxanthin, and lutein are the major antioxidants in fruits and act as scavengers of free radicals. Lycopene, lutein and astaxanthin and the colorless carotenoid precursors phytoene, phytofluene have also been associated with a decreased risk of cancers including prostate cancer [39–41], colon [42], and lung [43]. These benefits, of a carotenoid rich diet, could have a significant contribution to human health and previous authors have suggested that manipulating their metabolism could contribute to this goal [44].

**Fig. 1:**
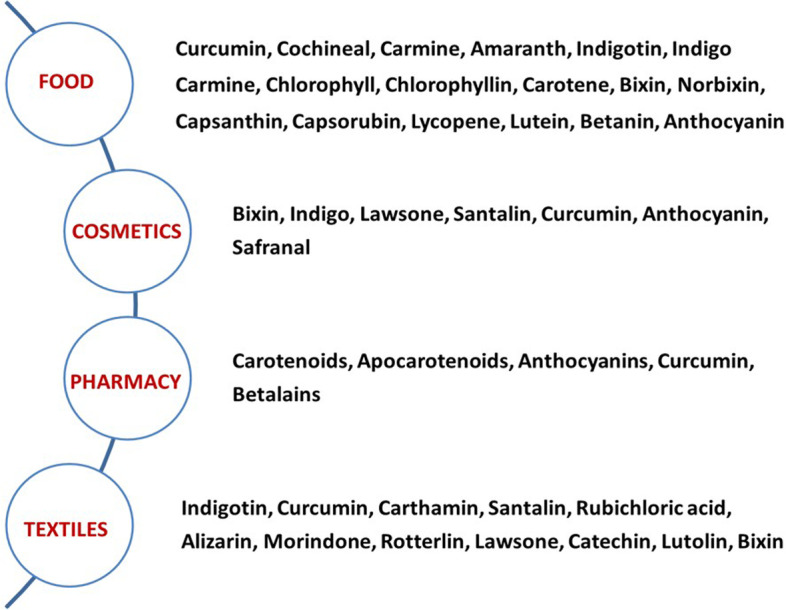
Industrial use of plant-based pigments

Among the carotenoids and their cleavage products, the highest oxidation potential of 0.94 V has been reported for bixin, an apocarotenoid, obtained from the seed arils of *Bixa orellana* [45] and thereby possess tremendous industrial potential (Fig. 1). Bixin has also been described as having anti-cancer properties towards osteosarcoma, breast, colon, prostate, anaplastic thyroid, and papillary thyroid cancers [46] as well as various potent pharmacological activities, including anti-inflammatory and antioxidant properties and is a promising candidate for the treatment of multiple sclerosis due to its ability to prevent neuroinflammation in mice primarily by scavenging ROS [47].

Berries have also been found to be richest source of anthocyanins among fruits, and blue berries have been reported to be the richest source of antioxidants with a TEAC (Trolox equivalent antioxidant capacity) value of 14.98 mM Trolox/100 g of dry weight due to high levels of proanthocyanidins and anthocyanidin [48].

Apart from their health benefits, pigments extracted from fruits have been found to have numerous applications as food colorants, in cosmetics, textiles & pharmaceutical industry [49] (Fig. 1). Considering the multifold applications of pigments, several extraction techniques ranging from conventional Soxhlet to the usage of novel techniques like electric field extraction have been designed to extract the desired pigments in optimum quantity and quality from fruits at various developmental stages of ripening (see Table 2).

**Table 2:** Extraction techniques of pigments from fruits

Pigment	Fruits	Pigment extracted	Extraction Method	Reference
CAROTENOIDS	Metabolite Profiling: matrix-assisted laser desorption ionization time-of- flight mass **s**pectrometry (MALDI/TOF-MS)**,** High pressure liquid chromatography (HPLC)			[50] [51]
	Tomato Gac Fruit Peel	Lycopeneβ-carotene, lycopene, and lutein	Atmospheric liquid extraction and maceration	[52] [53]
	Apricot, Peach and Tunisian Kaki	β-carotene, β-cryptoxanthin, lutein, zeaxanthin	Accelerated solvent extraction	[54]
	Carrot	β-carotene	Microwave- assisted extraction	[55]
	Tomato	Lycopene	Enzyme-assisted extraction	[56]
	Pomegranate	β-carotene, lutein	Green extraction	[57]
ANTHOCYANINS	Metabolite Profiling: matrix-assisted laser desorption ionization time-of-flight mass **s**pectrometry (MALDI/TOF-MS)**,** High pressure liquid chromatography (HPLC)			[58] [59]
	Blue berry, Cherry, red pear peel	glucosides, galactosides, rutinosides and arabinosides of delphinidin, cyanidin, petunidin, peonidin, malvidin,	Solvent extraction Ultrasound-assisted extraction	[60]
	Blueberries	glucodsides of delphinidin, cyanidin, malvidin,	Aqueous extraction method (Box-Behnken design)	[61]
	Figs	cyanidin 3-rutinoside	Heat, microwave, and ultrasound assisted extraction	[62]
	Blackberry	cyanidin-3-O-glucoside, cyanidin-3-O-rutinoside, cyanidin-3-O-6″ malonyl-glucoside, cyanidin-3-O-6″-dioxalyl-glucoside	Pressurized fluid extraction	[63]
CHLOROPHYLL	Metabolite Profiling: matrix-assisted laser desorption ionization time-of-flight mass **s**pectrometry (MALDI/TOF-MS)**,** High pressure liquid chromatography (HPLC)			[64] [65]
	Spinach	chlorophyll a, b, carotenoids	Electric Field and enzyme assisted extraction	[66] [67]

Among the several pigments present in fruits the major pigments such as chlorophyll, carotenoids, and anthocyanins possess diverse pigment functionality as they are essential for photosynthesis, possess multiple bioactivities, potent antioxidants, therapeutic properties, and industrial use, therefore an in-depth analysis of the factors regulating their biosynthesis, metabolism and storage during ripening is inevitable. This will further aid in designing biotechnological tools to enhance the production of these highly beneficial pigments in fruits.

## Regulation of pigment dynamics in fruit ripening

Pigment change during fruit ripening is a tightly controlled phenomenon signaled by plant growth hormone, several transcription factors, gene families, enzymes of the pigment biosynthetic pathways, and environmental stimuli [68]. Also, signaling molecules such as nitric oxide, melatonin, hydrogen sulphide [69] and sucrose have been highlighted for their role in accumulation of pigments during various fruit ripening stages (for details, see review by [70]). The ripening of fruit results in color change owing to pigment biosynthesis, degradation, and sequestration with the aid of development of new organs such as fibrils in pepper and plastoglobules in other fruits [71, 72]. Also, the pigments undergo several biochemical changes post synthesis to enhance their stability leading to the production of novel cleaved pigment products with probable bioactivity. In addition, mutant studies in the last two decades have uncovered the role of the regulatory mechanisms and have provided a platform to enhance the production of desired pigments with biotechnological tools [73].

### Manipulation of fruit de-greening enhances carbon yield

**Fig. 2:**
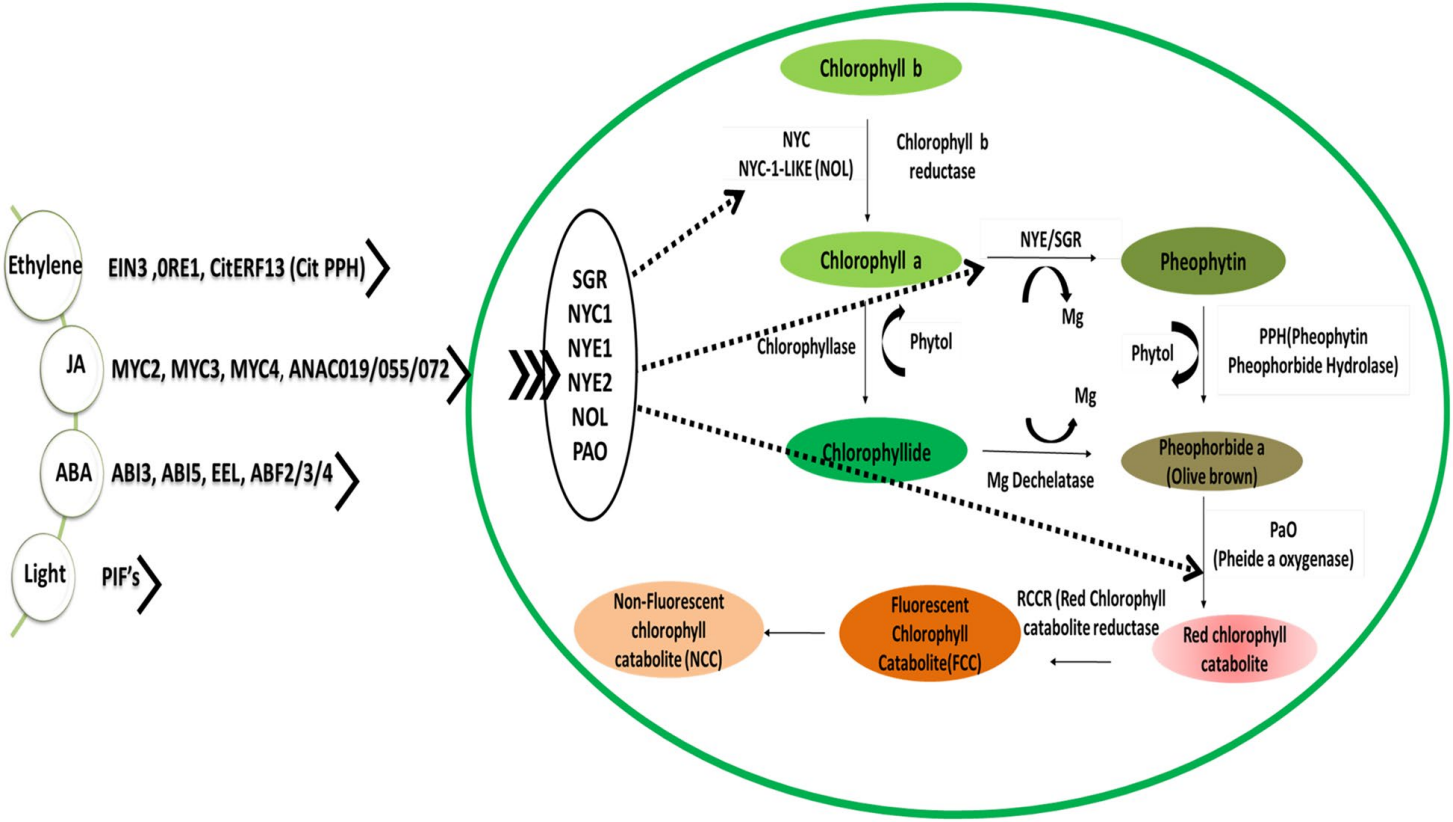
Regulation of chlorophyll degradation in fruit ripening. The figure describes the regulatory mechanisms (growth hormones and light) which signal specific transcription factors followed by activation of genes which initiate chlorophyll degradation. Ethylene signaling the following TF^s^: EIN3(ethylene insensitive 3),0RE1(a NAC transcription factor), CitERF13 (citrus ethylene response factor 13), ABA signaling the following TF^s^: ABI3, AB15(abscisic acid insensitive 3 and 5), EEL (b ZIP family), ABF2/3/4 (ABA)-responsive element (ABRE)-binding transcription factors), jasmonic acid signaling the following TF’s: MYC2 (basic-helix-loop-helix (bHLH), MYC3, MYC4, ANAC019/055/072(a NAC transcription factor). The TF^s^ regulated by light signaling are: PIF4 (phytochrome-interacting factor 4) and PIF5 (phytochrome-interacting factor5). The binding of the above stated transcription factors to the gene promoter sites (genes mentioned in the oval shape) enhances their activity and function. The genes mentioned are *SGR (stay green), NYC1(non-yellow colouring1), NYE1 (non yellowing1), NYE2, NYC1-LIKE (NOL) and PAO.* CitERF13 have been reported to bind to the promoter of PPH, respectively

As the fruit begins to ripen, the degradation of the green pigment chlorophyll, is initiated to promote remobilization of nutrient and promote biosynthesis of vitamins. The de-greening of fruits is important to promote detoxification of chlorophyll released from its binding proteins [74]. Light, along with the growth hormones ethylene, ABA and jasmonic acid, signal specific transcription factors (TF^s^) (see Fig. 2), which activate the functioning of the *CCG* (*chlorophyll catabolic genes)* involved in chlorophyll degradation [75, 76]. However, any mutation in the enzymes or signaling by the regulatory TF^s^ could lead to a stay-green phenotype, which has recently been reviewed by Zhu et al. [74]. However, slowing down of chlorophyll degradation is an effective strategy to enhance the fruit quality as it extends the time period of photosynthesis in the developing fruit, enhances the assimilation of carbon, soluble solid content and nutraceutical composition of fruits [77]. Several reviews have evaluated the role of fruit photosynthesis in carbon gain [10, 78–81] and the link between photosynthesis and the formation of key vitamins [82]. Besides, an insight into the regulation of chlorophyll degradation pathway has enabled food technologists to enhance the storage and shelf life of food by delaying de-greening with effective use of several chemical compounds like 1-MCP (1-methyl cyclopropene) [83] elevated carbon dioxide, [84], melatonin [85], and chlorine dioxide [86] resulting in inhibition of signaling by growth hormones, and suppression of genes which promote chlorophyll degradation (see Fig. 2). However, there are many fruits which remain green even when ripe, like some varieties of apples (Granny Smiths, Crispin), pear, green grapes, limes, guava, and cucumber, to name a few. “Stay green even when ripe” phenomenon has been attributed to suppression of genes which encode the chlorophyll degradation enzymes [87] and insensitivity to ethylene as shown in *Nr* (never ripe) mutants in tomato. The variations of color in tomato clarifies that regulation of carotenoid biogenesis is unique for each species. Recently, *Solanum habrochaites* (SH; green -fruited) was studied to decipher the molecular reasoning for green color retention. i) In SH a shift towards the β-carotene branch of carotenoid biosynthesis was found missing and both α- and β (carotene) branches were found to make equal contributions, ii) SH fruits were found to be insensitive to ethylene induced carotenogenesis, as in spite of emitting high levels of ethylene, they remained green, iii) SH fruits were found to retain the proteins related to photosynthesis and were lacking in proteins involved in conversion of chloroplasts to chromoplasts, iv) lack of carotenoid accumulation in spite of uncompromised expression of chromoplast specific genes such as *PSY 1(phytoene synthase1)* and *LCYB (lycopene β-cyclase)* in SH due to probable blocking by SNP’ (single nucleotide polymorphisms) resulting in lack of abundance of key proteins PSY1 and LCYB, v) diminished levels of homologues of fibrillin (FIB) like PAP3 (plastid lipid-associated protein) and CHRC (chromoplast-specific carotenoid-associated protein) involved in sequestration [88] However, carotenoid biosynthesis is independent of chlorophyll retention and is carried out in both red (normal pace) and green (slow and delayed) phenotypes with the green lines showing the presence of both the thylakoid and the plastoglobuli in the same plastid [89].

### Chloroplast to Chromoplast: accumulation of starch and carotenoids

**Fig. 3:**
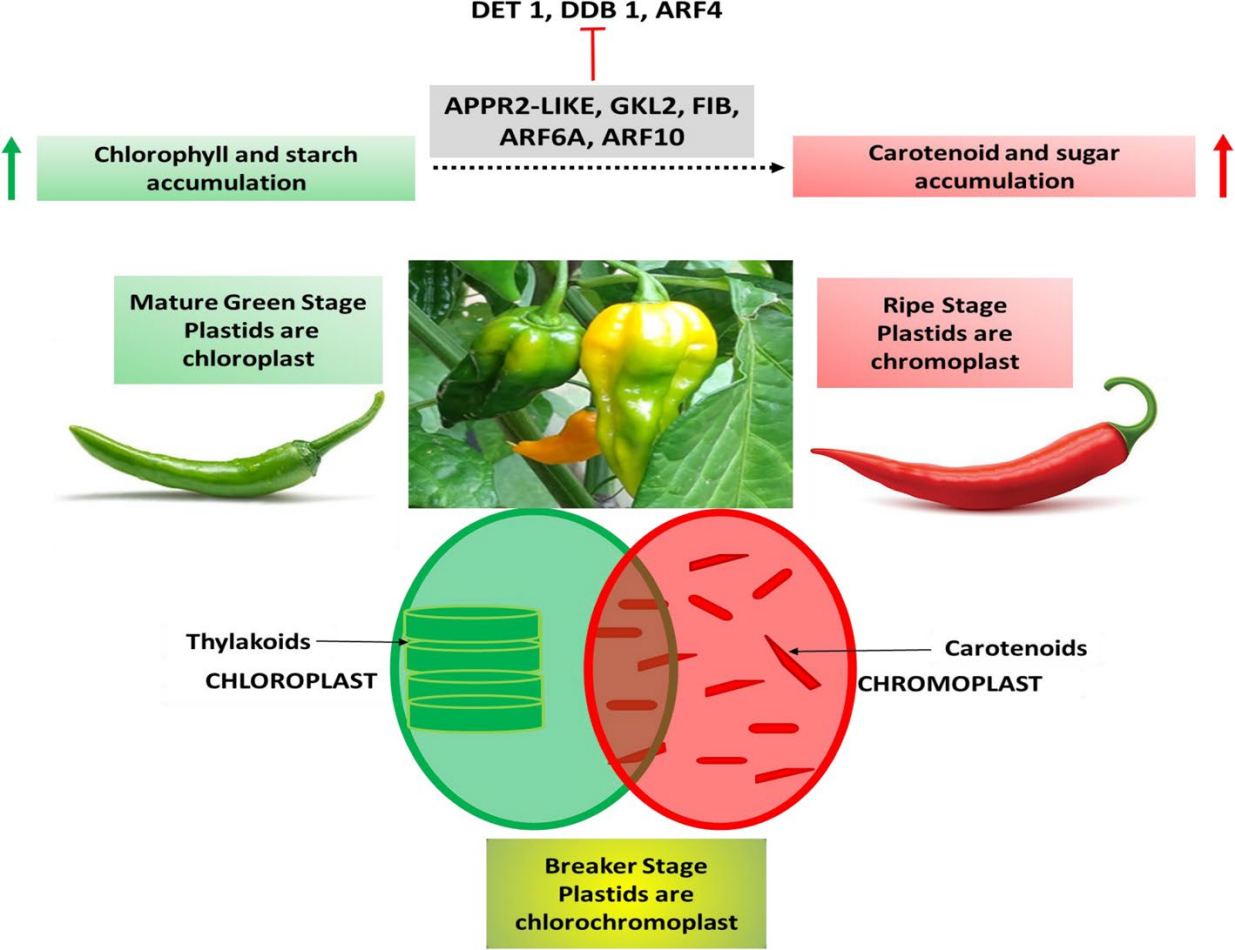
Regulation of transformation of chloroplast to chromoplast. The arrow (↑) indicates that an enhanced chlorophyll and chloroplast development results in augmented carotenoid and sugar content. TF^s^ DET1, DDB1 and ARF4 down regulate the conversion of chloroplast to chromoplast while APRR2-LIKE, FIB, GKL2, ARF6A, ARF10 upregulate the conversion

The degradation of chlorophyll in fruits like tomato and pepper is accompanied by a well-regulated conversion of chloroplast into chromoplast (see Fig. 3), accumulating carotenoids and result in a visible color difference from green to yellow to orange to red [90]. Carotenoids are sequestered in plastids (chloroplast and chromoplast) at high levels. In the chloroplast xanthophylls are produced for their photosynthetic utility in the thylakoid membrane [91]. However, during ripening, breakdown of the thylakoid takes place coupled with accumulation of carotenoids like lycopene in the membrane along with synthesis of membranes as sites for carotenoid biosynthesis, coupled with increased number and size of plastoglobules [72, 92]. In the chromoplasts, the plastoglobules are highly enriched with esters of carotenoids and enzymes involved in carotenoid metabolism (for details see review [93]). The FIBs play a crucial role in development of plastoglobules and fibrils in fruits for storage of carotenoids. In a trial by Simkin et al. [72] the role of *FIB* gene was assessed and it was found that it delayed thylakoid loss in differentiating chromoplast and resulted in an increase in plastoglobuli number and thereby increased the concentrations of carotenoids like β-carotene and lycopene [72].

The chlorophyll content of an unripe fruit such as tomato has been found to be directly linked to the sugar content of the ripened fruit, thereby affecting the nutritional quality and taste of a ripened fruit. Therefore, the regulatory mechanism involved in chloroplast development and accumulation of chlorophyll are crucial. DET1 (De-etiolated 1) and DDB1 (UV-damaged DNA-binding protein1) control chloroplast number, size and development as negative regulators [94], Also GOLDEN2 LIKE TF^s^ GLK1 and GLK2 play a crucial role in chloroplast development and accumulation of chlorophyll [95] along with its homolog APRR2-LIKE (Arabidopsis pseudo response regulator2) [96] (see Fig. 3). The transcriptional activation of GLK 2 and *APRR2-LIKE* genes is carried out by TKN2 and TKN4*,* the two KNOX (Class I KNOTTED1-LIKE HOMEOBOX) proteins [97]. In addition, ARFs (auxin response factors) are involved in transcriptional regulation of fruit ripening by repressing or activating transcription of auxin responsive genes. In tomato, ARF4 has been reported to negatively regulate chlorophyll accumulation in the fruit along with starch biosynthesis. A reduced ARF4 content resulted in a dark green fruit with enhanced chlorophyll, increased chloroplast number accumulated more starch at early stages (green fruit) resulting in more sugar and higher soluble solids at ripening stages (excess starch broken down into sugars) [98]. While ARF6A content was found to be directly proportional to increased chloroplast, chlorophyll, rate of photosynthesis and sugar accumulation as it bound to promoter of GLK1 and positively regulated its activity [99]. Similar findings have been reported for ARF10 being an activator of GLK1 and thereby promoting chlorophyll accumulation [100]. An overexpression of GLK2 has been found to increase the total carotenoid content, sugar and fruit starch [101]. Also, in tomato, with the development of chromoplast the genes for lycopene synthesis are upregulated [102], while the enzymes involved in lycopene metabolism such as lycopene-e-cyclase (LCYE) and LCYB are downregulated [103]. Therefore, at the ripe stage, chromoplasts are completely developed and become a reservoir of carotenoids and thereby appear red.

With the development of chromoplast the carotenoids which would determine the final fruit color are accumulated and the predominant carotenoid shape up the final form of its deposition which is crucial for color, bioavailability, and stability of the carotenoid. This phenomenon has been demonstrated for fruits of *Physalis pubescens* L. (yellow), *Physalis peruviana* L. (orange), and *Physalis alkekengi* L. (red) wherein the yellow and orange fruit of *Physalis* showed gradual accumulation of β-carotene and lutein at varied concentration, justifying their different hues on ripening. However in the red fruit of *Physalis* (on being fully ripe) traces of β-carotene, and high levels of β-cryptoxanthin and zeaxanthin were reported [104]. Therefore, the development of chromoplast not only initiate value-addition of color tones but enhance the nutritional efficacy of the ripened fruit. Also, for the fruits which “stay green when ripe” there are prospects for conversion of chloroplast to chromoplast and enhancement of nutritional value synthetically by the overproduction of phytoene with the crtB enzyme (bacterial PSY enzyme) as demonstrated recently in leaf chloroplasts resulting in reprogramming of plastid-nucleus interactions leading to development of chromoplast and subsequent carotenoid biosynthesis [105]. The regulation of chromoplast biogenesis is beneficial for both biosynthesis and storage of carotenoids (For details see review [106]). Therefore, research on chromoplast differentiations channelizes development of fruits with augmented carbon yield and sugar when ripe along with enhanced production of carotenoids with multifold health benefits.

It has been shown on several occasions that secondary metabolism and, particularly, plastid metabolism is not only transcriptionally but also post-transcriptionally regulated. Recent developments in technologies based on mass-spectrometry has paved the way for a deeper coverage of protein expression. This has shifted the focus on translational and post translational protein expression in tomato and other fruits, which is currently limited to transcription, thereby adding a novel dimension to the already existing data on transcriptomic, metabolomic and genomic resources [107]. The role of Clp protease in chromoplast development and carotenoid accumulation in tomato fruit ripening has been investigated recently. These researchers concluded that Clp protease work in coordination with specific chaperones that lessen the protein folding stress, enhance the stability of enzymes involved in carotenoid accumulation and inhibit carotenoid degradation [108]. In a yet another trial, CHLORAD (chloroplast-associated protein degradation proteolytic pathway in transition of chloroplast to chromoplast has been reported. These researchers emphasize on the crucial role of chromoplasts in fruit ripening and suggest strategies for bioengineering based crop improvements [109].

### Carotenoid biosynthesis pathway: a reservoir of nutraceuticals

**Fig. 4:**
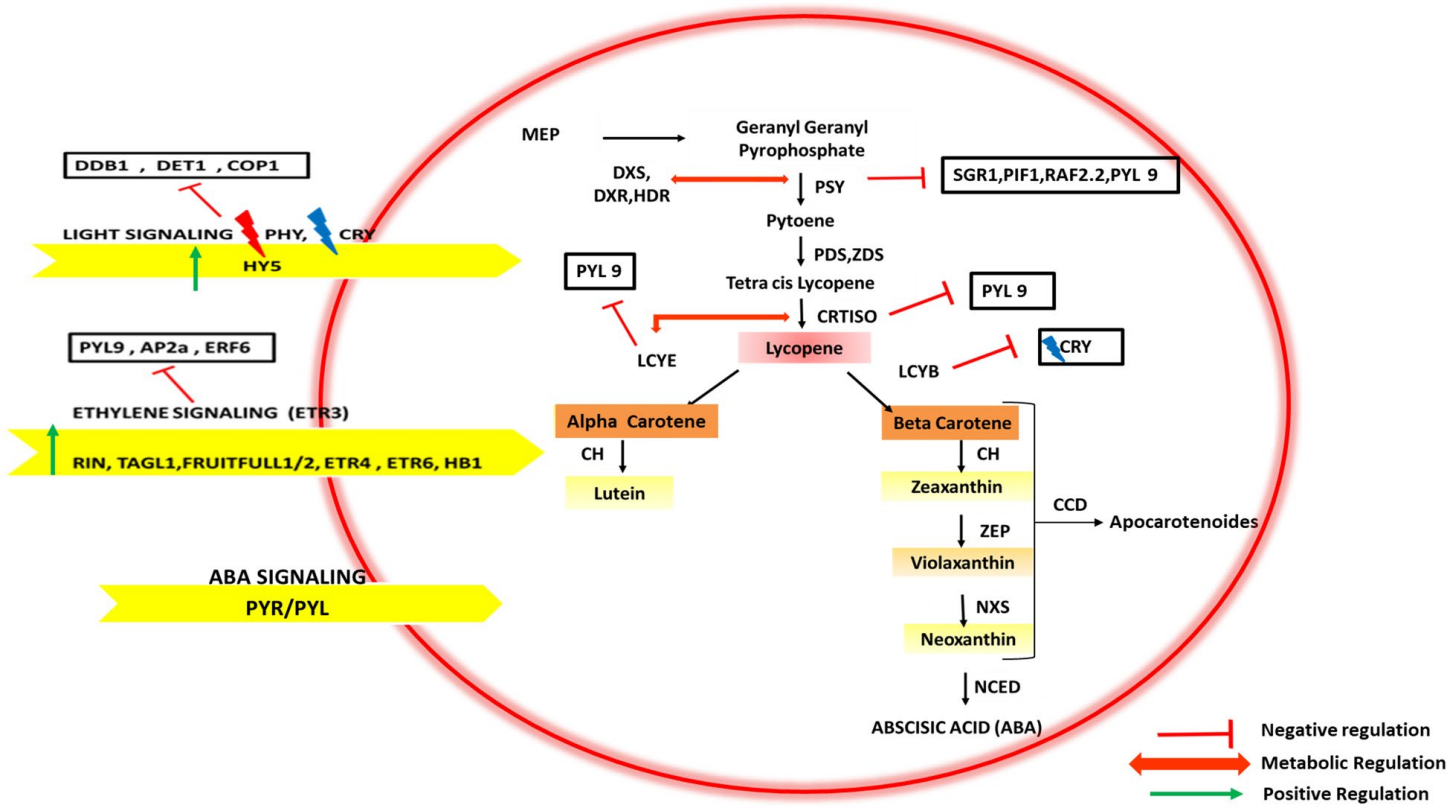
Biosynthesis and regulation of carotenoids. The red oval indicates the carotenoid biosynthetic pathway in the chromoplasts. The yellow arrows indicate the regulatory mechanisms initiated by ethylene and light which upregulate carotenoid biosynthesis. However, the ABA signaling mediated by PYL 9 inhibits lycopene accumulation

The expression of carotenoid pigment is regulated at three important stages: biosynthesis, degradation, and lipoprotein sequestering structures, respectively. The regulation of carotenoid biosynthetic pathway in fruit ripening (see Fig. 4) is multifold and carried out via signaling by plant growth hormones, enzymes of the pathway and environmental stimuli such as light. The network of TF^s^ and growth hormones such as ethylene and ABA play a critical role in activation of ripening genes. Together they regulate accumulation of carotenoids (in chromoplast) and anthocyanins (in vacuoles). Among the MADS BOX family genes positively regulating ethylene signaling and carotenoid biosynthesis *ripening inhibitor (RIN)* is reported as the master regulator. Studies on mutants *ripening-inhibitor (rin), non-ripening (nor),* and *Colorless-nonripening (Cnr*) have led to novel findings in the ripening process of climacteric fruits like tomato [110]. It has been proposed to generalize physiological attributes in these three mutants; i) in all development occurs up to the mature green stage, with full size fruit yet do not proceed to ripening, ii) all fail to emit ethylene associated with ripening and are unresponsive to exogenous ethylene, however iii) they retort to ethylene in other fruits and tissues induced with ethylene responsive genes. To sum up, these features indicate that all three mutations influence the phenomenon of ripening, essential for ethylene induction along with activities which cannot be compensated by ethylene alone and therefore all three genes are probably conserved regulators influencing even non-climacteric fruit ripening. Recently, fruits deficient in *RIN* (generated by CRISPR/Cas9) led to partial ripening with only 10% concentration of ethylene and carotenoids in comparison to the wild type [111]. Other transcription factors initiated by ethylene signaling to positively regulate carotenoid biosynthesis are TAG1 (tomato agamous 1), TAGL1 (tag-like1), FRUITFULL1/2, ETR1(ethylene receptor 1) ETR6 (ethylene receptor 6) and HB1 (HD Zip homeobox protein) (see Fig. 4). On the contrary, PYR/PYL9 (pyrabactin resistance/ pyrabactin resistance - like), AP2a (apetala2), ERF6 (ethylene response factor 6) are negative regulators of ethylene signaling. Along with ethylene, the regulatory role of ABA in carotenoid biosynthesis by exercising control over plastid development has been emphasized [112]. As per recent findings of Kai et al. (2019), ABA signaling is mediated by its promoter PYR/PYL9*,* which regulates the quantity and quality of carotenoid in fruit ripening [113]. These researchers highlighted that in comparison to wild type, over expression of PYL9 lead to on early ripening onset (due to excessive ABA accumulation at mature green stage simultaneously inducing release of ethylene), followed by maximum ABA accumulation at breaker stage resulting in a drop in ethylene levels due to negative crosstalk between ABA and ethylene, thereby affecting fruit color [113]. Furthermore, the crucial carotenoid biosynthetic genes, encoding ethylene regulated *PSY1,* and the other enzymes like carotene isomerase (CRTISO) and LCYE were reported to be regulated negatively by PYL9 [113]*.* In contrast, LCYB was positively regulated by PYL9, thereby limiting the entry of metabolites in the lycopene pathway and channeling the carbon towards β-carotene and ABA (similar findings were reported by [114]). These researchers confirmed that ABA signaling, mediated by *PYL9,* regulates the genes related to release of ethylene, metabolism of pigment and degradation of the cell wall [114]. Also, curbing the expression of *NCED1*, which is a vital gene for biosynthesis of ABA, upregulated the production of ethylene and PSY1 and negatively regulated the production of LCYB resulting in enhanced levels of β-carotene and lycopene [115]. Along with the regulatory action of growth hormones the enzymatic regulation plays a critical role in carotenoid accumulation. The over expression of enzymes like DXS (deoxy xylulose5-phosphate synthase) and DXR (deoxy xylulose5-phosphate reductoisomerase) involved in regulating the carotenoid flux have been reported to increase the concentration of carotenoids [116] and similar findings have been reported for the other key regulatory enzymes PSY and CRTISO [117]. The *PSY* genes are sensitive to periods of stress, scarcity of water, excessive light, ABA, salinity, and fluctuations in development of the plant. There are three isoforms of *PSY* genes reported in grasses (*Poaceae*) [118] and fruits like tomato possess *PSY1* in fruits, *PSY2* in leaf tissues and *PSY3* in roots under stress [117, 119]. In maize roots, *PSY3* expression was induced in periods of stress like drought leading to an increase in the production of ABA and escalation of the carotenoids [118]. In addition, as per *recent findings it has been suggested that the PSY gene family compensates for the loss of PSY1 as reported in a yellow pepper variety,* Micro Pep Yellow [120].

The *PSY* genes are positively regulated by transcription factor RIN and negatively regulated by SGR1, PIF1 and RAP2.2 [90]. A light-induced transcription factor RAP2.2, has been reported to bind to *PSY* gene promoter site, thereby leading to an altered expression of the pigment [121]. The genetic regulation of *CRTISO* gene is carried out by SDG8 (*histone methyl transferase enzyme)*, responsible for methylation of chromatin linked with *CRTISO* gene. The absence of SDG8 leads to reduced gene expression and resultant decline in the biosynthesis of lutein and strigolactones [122]. A *CRTISO* mutant *ccr1*, has been reported to downregulate more than 80 genes along with *CRTISO* gene. Another *CRTISO* mutant *ccr2* was found to stimulate increased production of cis-carotenes in chromoplasts [122]. In addition, metabolic feedback regulation is carried out by the enzymes DXS (for *PSY*) and LCYE (for *CRTISO*) respectively. This phenomenon has been observed in etiolated Arabidopsis seedlings where an elevated expression of *PSY* increased carotenoid levels due to DXS accumulation, which indicates metabolic feedback regulation triggered through *PSY,* which in turn enhanced the availability of MEP substrates [123].

Apart from enzymes, there are several environmental factors which are involved in regulation of carotenoid biosynthesis, among them, one of the crucial factors is light. Photoreceptors like PHY (phytochromes) are sensitive to red light and enhance the activity of *PSY* while CRY (*cryptochromes*) are sensitive to blue light and enhance the levels of pigments like chlorophyll and anthocyanin in leaves, as well as lycopene in fruits along with a decline in LCYB expression [90]. In tomato, PHY as temperature sensors have been reported to affect plastid metabolism in the leaves and tomato fruit and the accretion of isoprenoid derived compounds. The studies on triple mutants *phyAB1B2* (phytochrome silenced plants) demonstrated that the biosynthesis of the major carotenoid lycopene was found to be sensitive to PHY-temperature perception [124].

The light signaling is regulated positively by HY5 (elongated hypocotyls) and negatively by DDB1, DET1 and COP1 (constitutive photomorphogenic1) (see Fig. 4). The PIF1 (phytochrome interacting factor 1) regulates the signaling induced by light by functioning downstream and act as a negative regulator of phytochromes [125]. Recently a self-shading model for regulation of carotenoid biosynthesis by PIF has been showcased. These investigators reported that in an unripe green fruit, when sunlight passes through its flesh, high levels of PIFs are maintained by a self-shading effect which in turn downregulate the initiation of carotenoid biosynthetic pathway and thus prevent carotenoid production. However, with the onset of chlorophyll degradation resulting in breakdown of PIFs the self-shading effect weakens thereby activating the carotenoid biosynthetic pathway as fruit ripening proceeds. These researchers also suggest strategies to manipulate this mechanism to obtain fruits enriched with carotenoids [126]. Furthermore, exposure to UV (UV-B/C) radiations enhances the pigment concentration and the activity of genes encoding various enzymes involved in the biosynthesis pathway which imparts strength to the plant to survive in periods of stress [127].

In addition to the natural regulatory mechanism of the plant, selected carotenoid targeted regulation has been made possible by genetic engineering models to enhance the content of a specific carotenoid in the fruit. The biotechnological interventions for transgenic metabolic engineering, multigene engineering (gene scissor: CRISPR/cas9) and genetic breeding, in the last two decades have made it possible to enhance the productivity, nutritional adequacy and economic value of fruits [128] with enhanced carotenoids like lycopene [129], β-carotene [130] zeaxanthin, (enhanced to make up for 50% of the total carotenoid present in the fruit) [131], lutein and neoxanthin. The enhancement of these pigments (like β-carotene) initiates carotenoid biofortification, which can be further enhanced by downregulating the enzymes involved in carotenoid degeneration while enhancing carotenoid stability and retention [132].

### Altered carotenoid chemistry in chromoplast to enhance stability

In horticultural crops, carotenoids accumulate in chromoplasts, which could vary from crystalline (carrot, papaya, tomato), globular (mango), fibrillary (pepper) [133] membranous (watermelon, mango, butternut squash), to reticulo-tubular based on sequestering structures in the chromoplasts [134]. In addition, more than one type of chromoplasts can coexist in a particular species of fruit. The specific carotenoid accumulation is based on pigment-bearing substructures as seen in the case of red tomato and watermelon with carotenoid crystals resulting in huge accumulation of lycopene [135, 136] and β-carotene in carrot, [137] and orange cauliflower [138].

Several complex biochemical changes take place in the chromoplast leading to transformation of previously synthesized pigments and de novo biosynthesis of new pigments. In fruits, carotenoids are subjected to many chemical reactions like oxidation, epoxidation, (cis-trans) isomerization and cleavage of polyene chains. To increase the carotenoid accumulation in plant cells esterification is essential. Recently, several esterases XES (xanthophyll esterase) have been identified in plants [71, 139]. The esterification of violaxanthin and neoxanthin was found to be carried out by an XES homolog PYP1 (pale yellow petal 1) in tomato [140], while in wheat, XAT (xanthophyll acyl-transferase) catalyzed the esterification of lutein [141].

Carotenoids undergo esterification with fatty acids which makes them fat soluble and also facilitate their build up in chromoplast [142]. The yellow xanthophylls are less stable due to their esterification by myristic and linoleic acid, which are unsaturated fatty acids. A pinacolic reordering of the epoxy-cyclohexenyl groups in the chromoplast membranes converts epoxy-xanthophylls, like violaxanthin and antheraxanthin, into keto-xanthophylls like capsanthin, capsorubin and cryptocapsin [143].

In contrast to yellow xanthophylls, the red xanthophylls like capsanthin and capsorubin, found in ripe pepper fruit, demonstrate more stability owing to their esterification by lauric and palmitic acid which are short chain fatty acids. The augmented carotenoid stability by esterification could aid in biofortification and thus be explored in depth by future research projects [132]. In addition, the report on the cloning of the pepper enzyme, CCS (capsanthin/capsorubin) [144] and its promoter [145], provides a model to alter the biosynthesis and metabolism of cyclic carotenoids in chromoplasts of pepper fruits and the re-engineering of carotenoid biosynthesis in other carotenoid accumulating fruit.

The chemical changes in the chloroplast lead to the accumulation of keto-carotenoids with changes in metabolism of lipids as galactolipids levels have been found to diminish while phospholipids have been reported to accumulate in chromoplasts. Oxygenated configurations of carotenoids having acylcyclopentanol end groups like capsanthin, capsanthin-5,6-epoxide and capsorubin have been observed to control the intensity of red color in chili pepper fruits [146]. In ripened pepper fruits, ketoxanthophylls (capsanthin, capsorubin and capsolutein) accumulate the most, followed by xanthophylls (violaxanthin and zeaxanthin) then the epoxyxanthophylls (capsanthin-3,6 epoxide and capsanthin-5,6-epoxide) along with a small accumulation of lycopene. Due to several chemical reactions in the chromoplast, many novel carotenoids have been detected in ripened paprika fruits like 3′-deoxy-capsanthin and 3,4-dehydroxy-3′-deoxycapsanthin [147].

### Carotenoid metabolism and storage in fruit ripening

The ripening of the tomato fruit has been investigated recently for the coordinated regulation of the genes involved, chromatin, epigenetics, transcriptomics, post translational and at the level of protein expression. These investigators have reported that the non-coding RNAs play a crucial regulatory role in fruit ripening at transcriptional and post transcriptional levels (for details see review [148]).

The final concentration of carotenoids in plant tissues is dependent upon the enzymes; lipoxygenases, and CCDs (carotenoid cleavage dioxygenases) involved in degradation and cleavage. In Arabidopsis four *CCD* genes namely *CCD1, CCD4, CCD7* and *CCD8* and five NCEDs (*9-cis epoxy carotenoid dioxygenases)* namely *NCED2, NCED3, NCED5, NCED6* and *NCED9* have been identified [44, 149]. Many trials on fruits report that the carotenoid content in fruits and the expression of *CCD1* and *CCD4* are inversely proportional to each other [150]. Also, while *CCD1* is closely related to production of volatile compounds like aroma in fruits, *CCD7* and *CCD8* play a vital role in strigolactones production [151–153] and the *NCED* family plays a major role in production of the growth hormone ABA [154].

CCDs cleave the carotenoids into apocarotenoids and majority of them like, bixin, saffron, crocin, apocarotenals, apolycopenoides, ionones (i.e., β-ionone), peridinin and many more have been reported as bioactive compounds with therapeutic efficacy and industrial applications [44, 155–159]. CCDs play an important regulatory role and impact the final content of carotenoids and the presence of volatile compounds which affect flavor and aroma. For example, β-carotene, the precursor of retinal (Vitamin A), is the immediate precursor of one of the most important flavor volatiles, β-ionone [160]. In tomato, two enzymes, CCD1A and CCD1B, that can cleave multiple carotenoid substrates to generate geranylacetone, pseudoionone, and β-ionone have been identified [161–163]. CCD’s have also been reported to have a role in controlling the fruit color. In peach, a mutations in *PpCCD4* attenuates its levels resulting in yellow flesh variety due to truncated protein with reduced carotenoid degradation activity [164, 165].

In citrus fruits (*Citrus unshiu*), cleavage of β-cryptoxanthin and zeaxanthin by CitCCD4 results in the formation of β-citraurin, which is responsible for the reddish color in the peel. The production of C_30_ apocarotenoid in orange has been linked to CCD4b, thereby influencing the carotenoid accumulation in citrus fruits [166, 167].

Chromoplasts act as metabolic sinks to sequester the biosynthesized carotenoids into carotenoid-lipoprotein structures such as fibrils and plastoglobules [168]. These structures help to accumulate carotenoids and prevent overloading and inhibition of carotenoid synthesis [133]. The variability in these structures is linked to the variations of carotenoid profile in various fruits therefore governing these structures would directly influence carotenoid accumulation. The carotenoid associated proteins, fibrillin in pepper and its orthologue, carotenoid-associated protein (CHRC) in cucumber [169] play an important role in the production of carotenoid-lipoprotein structures [170]. In tomato, fibrillin overexpression has resulted in enhanced carotenoids and volatiles production [72].

Carotenoids are produced in various plastids but accumulate in chromoplast at high levels resulting in attractive hues of fruits thereby the regulation of chromoplast biogenesis has a strong impact on the carotenoid biosynthesis and accumulation. At the molecular level the *Or* (Orange) gene has been reported to initiate biogenesis of chromoplasts, as its mutants have been reported to initiate non colored plastid differentiation into chromoplasts with an augmented ability to amass β-carotene in potato, cauliflower [171] and melon [172]. Recent developments in RNA-Sequence transcriptomic profiling and microscopic analysis revealed the impact of overexpression *Or* gene resulting in augmented chromoplast size in very young fruits, promoted flower and fruit development, enhanced ethylene production, expression of ripening associated genes a [173]. Thus, *Or* gene has been a much sought-after target for nutritional biofortification and alteration of horticultural traits in agricultural products [44, 173, 174]. The modification of *Or* gene by genome editing has been proposed to manipulate carotenoid biosynthetic pathway, thereby making use of its interaction with PSY resulting in enhanced sequestering, protection from degradation and improved plants tolerance to abiotic stress [174].

Furthermore, the activity and accumulation of biosynthetic enzymes such as PSY was found to be regulated by chaperones *Or and Hsp70* in liaison with the Clp protease complex in the stroma, thereby suggesting that different chaperone families target distinct processes with regard to carotenoid accumulation and metabolism in fruit ripening [175]. Several biotechnological tools have been implemented to alter and enrich the plant tissues with carotenoids (see [44, 82]). However, most of these strategies have been focused on manipulating carotenoid biosynthesis and metabolism. Recently enhancement of carotenoid sink capacity in plastids by supporting the differentiation of carotenoid sequestering structures in plant tissues has gained much importance. The, over-expression of the Or protein, for example, promotes the formation of carotenoid-sequestering plastoglobuli in transgenic corn [176] and a 2-fold increases in carotenoids in transgenic cassava [44, 177]. Supporting evidence for the manipulation of deposition structures comes from work carried out by Simkin et al. [72]. The over-expression of the fibrillin protein in tomato resulted in an increase plastoglobuli number, and an increase in β-carotene (+ 64%) and lycopene (+ 118%) [72]. These authors also demonstrated that this increased pool of carotenoids resulted in a 36 and 74% increase in the β-carotene derived volatiles β-ionone and β-cyclocitral respectively and a 50 and 122% increase in the lycopene derived volatiles citral and 6-methyl-5-hepten-2-one respectively [72].

Furthermore, the transfer of carotenoid biosynthesis to the cytosol can be brought about by the activity of crtB in plastids and its combination with cytosolic bacterial enzymes to produce carotenoids in extraplastidial sites [178, 179] and accumulation of lycopene in the cytosol of tobacco [179]. The isoprenoid precursors are transferred to the cytosol via the MVA (mevalonic acid) pathway. These strategies have opened novel approaches for carotenoid biofortification and thus creating more space for carotenoids in plants (for details see reviews [174, 180]).

### Anthocyanin biosynthesis: enhances flavonoid content of ripened fruits

**Fig. 5:**
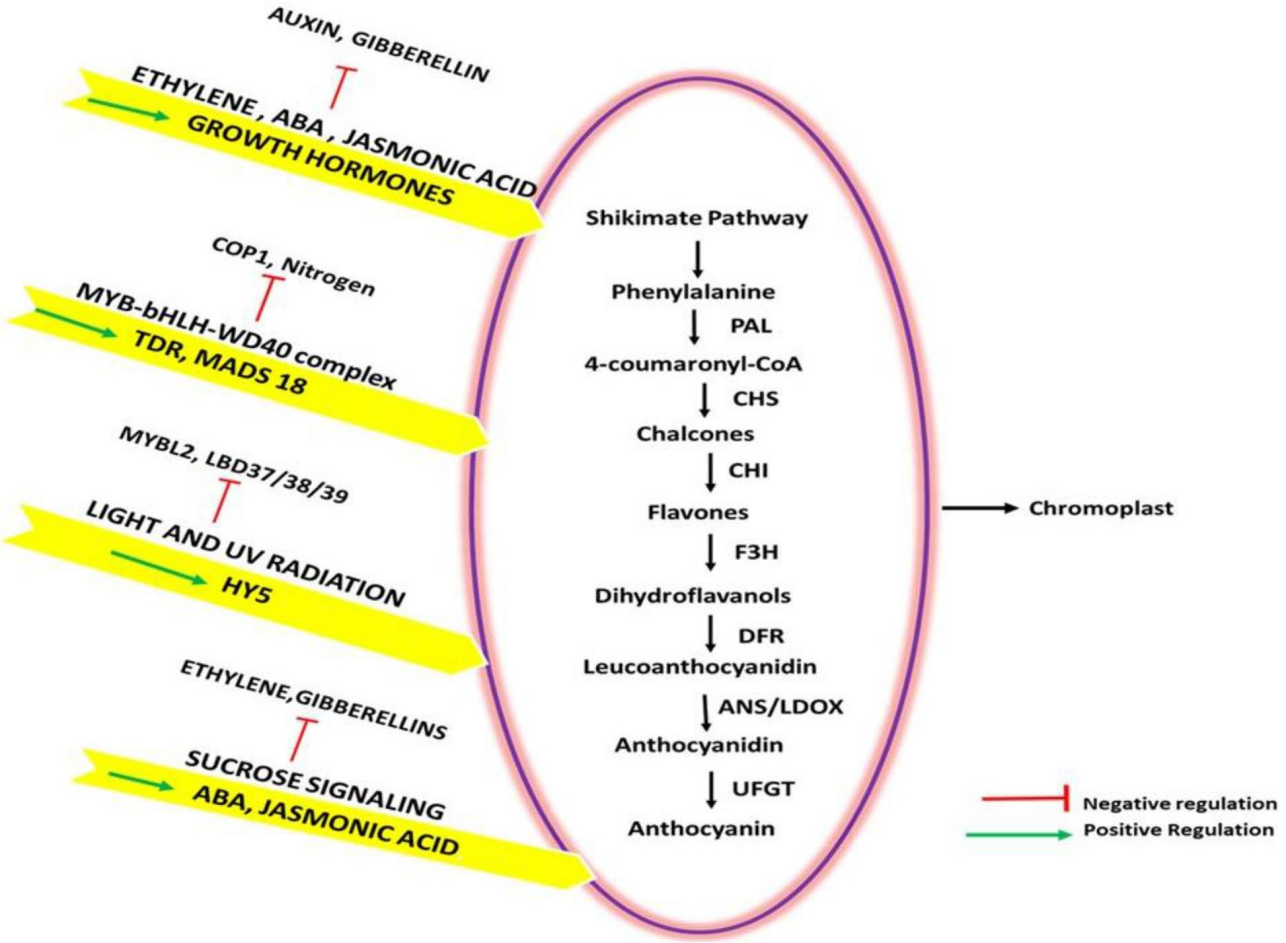
Regulation of anthocyanin biosynthesis in fruit ripening. The oval describes the anthocyanin biosynthetic pathway which is positively regulated by MBW complex, specific growth hormones, light, and sucrose signaling. In addition, the negative regulatory factors of anthocyanin biosynthesis have been mentioned. Auxin and gibberellin negatively regulate the signaling by the other growth hormones, while COP1 and nitrogen downregulate MBW complex. Also, MYBL2 and LBD37/38/39 are negative regulators of light signaling while ethylene and gibberellin downregulate the signaling of anthocyanin biosynthesis by sucrose

There are numerous examples of fruits and vegetables like pomegranate, cherry, plums, turnip, blackberries, blueberries, strawberries, red pears, onions, red cabbage, red apples, prunes, eggplant, cranberries and grapes which accumulate anthocyanin when ripe and change from green to purple or red [181]. Fruits exhibit varied pigmentation of anthocyanins in flesh and/or skin. As ripening proceeds, anthocyanin biosynthesis is triggered in some fruits post chlorophyll and carotenoid degradation [182]. In a trial on pomegranate, the development of red color was reported with an increase in anthocyanin pigment and degradation of chlorophylls and carotenoids. The major anthocyanins reported in the pomegranate (*Punica granatum* L.) peel were cyanidin 3,5-diglucoside and cyanidin-3-O-glucoside respectively [183]. Also, in ripened avocado (*Persea americana* Mill.), color changes from green to purple to black have been attributed to a fall in chlorophyll and an increase in glycosylated anthocyanin; cyanidin 3-O-glucoside [184]. The anthocyanins cyanidin-3-rutinoside and cyanidin-3-glucoside were also reported for ripening induced color changes in cherries (*Prunus avium*) [185]. The biosynthesis of anthocyanin is regulated by multiple factors like hormonal, genetic and environmental (see Fig. 5). An in-depth study of these regulatory factors involved in anthocyanin biosynthesis highlight the pathways for biotechnological editing to enhance pigment content. R2R3MYB, BASIC HELIX-LOOP HELIX (bHLH), WD40 COMPLEX are the three main TF^s^ regulating the structural genes of anthocyanin biosynthesis at an individual level and as a team as MBW complex. In a recent analysis *RrMyb10* was reported as the anthocyanin inducer in Ribes species, and its role in manipulating anthocyanin biosynthesis in heterologous systems has been highlighted [186]. Also, recent comparative analysis and genome-wide identification of MYB TF^s^ in two varieties of banana, *Musa acuminata* and *Musa balbisiana* would enable future prospects for functional studies [187]. Apart from these, *SEPALLATA* and *SQUAMOSA class MADS BOX* genes also regulate anthocyanin accumulation in fruits. In addition, anthocyanin biosynthesis can also be triggered by sucrose signaling by inducing *production of PAP1 (anthocyanin pigment1)* [188] along with the support of the plant growth hormones. Among the plant growth hormones, ABA and jasmonic acid act synergistically with sucrose signaling while gibberellin has been found to inhibit sucrose signaling [189]. Though ethylene signaling has been reported to enhance anthocyanin biosynthesis in several trials [190, 191], however, in Arabidopsis, ethylene inhibits anthocyanin accumulation which is induced by sugar and photosynthesis by curbing the activity of TF^s^ positively involved in regulation of MYB-bHLH-WD40 and increasing the expression of MYBL2 (a negative R3-MYB regulator) along with down regulation of SUC1 (*sucrose transporter 1)*, *PAP1, TT8 and GL3* [192]*.* In addition, auxins have also been reported to regulate anthocyanin biosynthesis in a negative manner [193, 194]. The role of ABA has been reported in a number of trials as a positive regulator of anthocyanin synthesis [195]. Silencing of key ABA biosynthetic gene *FaNCED1* and *LbNCED1* resulted in minimal anthocyanin production in strawberries and lyceum plants [196]. Among the non-climacteric fruits, strawberry has been considered as a model fruit to study ripening related characteristics, wherein both sucrose and ABA are involved in signaling anthocyanin biosynthesis. However, a recent trial on bilberry (*Vaccinium myrtillus* L.) (non-climacteric fruit) demonstrated that treatment of unripe bilberry fruits (attached/detached from the plant) with ABA resulted in anthocyanin accumulation and cell wall modification while the sucrose treatment of unripe bilberry fruits did not promote anthocyanin biosynthesis. Therefore, these results indicate that regulation of fruit ripening, is fruit specific and would differ from one fruit to the other [197].

Manipulation of both the functional genes and the TF^s^ involved in regulating anthocyanin biosynthesis have been found to enhance the flavonoid content. The silencing of *DET1* gene has resulted in reformed developmental processes mediated by light and an augmented flavonoid content [198]. Also, the flavonoid content of the tomato peel has been reported to be increased by 78 fold by overexpressing *chalcone isomerase (CHI)* gene in tomatoes [199]. However, the expression of two snapdragon genes *Ros1* and *Del* which encode MYB and *bHLH* resulted in the most substantial increase in anthocyanin content leading to development of fully purple hue on tomato fruits [200]. In addition, crossing the *Del/Ros1* lines with overexpressing lines of AtMYB12 resulted in enhanced accumulation of anthocyanin [201]. Furthermore, the anthocyanin accumulation genes *Del* and *Ros1* were recently transferred from transgenic Micro-tom to Moneymaker tomato cultivar via traditional breeding. The anthocyanin content in the inbred fruit enriched with anthocyanin was escalated to about 131% of the parent level, phenolic compounds upsurged by 51% coupled with an augmented antioxidant activity and reduced growth of bacteria [202]. Recently, in tomato, SlMYB75 has been reported to be effective in inducing anthocyanin accumulation at the rate of 1.86 mg/g in varied tissues, coupled with an increase in production of ethylene,flavonoid, phenolic compounds, and aroma [203]. Therefore, targeting of specific TF^s^ could engineer anthocyanin accumulation in a ripened fruit and thereby enhance its nutraceutical and industrial potential.

### Response of anthocyanins to stimuli suggests ways to enhance its production

Anthocyanins have been termed as “chameleons” or the “color diversity hub” due to frequent changes in expression of color related to biotic or abiotic factors like a drop-in temperature, brininess, dearth of nitrogen and minerals like phosphorous which are generally expressed as purple coloration on stem, leaves and other parts of the plant as response to low light stress. Anthocyanins display changes in tones due to environmental factors [204]. The soil pH affects the pH of cellular compartments thereby affecting the subcellular concentration of flavonoids and expression of pigments ranging from red to blue. Fertilizers rich in nitrogen have been reported as negative regulators resulting in a fall in PAP1 and TT8 proteins from WD40-bHLH-MYB complex, while they enhance the formation of negative regulators like LBD37/38/39 [205]. The role of temperature and light on anthocyanin accumulation has been well studied. In a trial on grapevine berries, changes in temperature varied the expression of the *MYB* genes (*VlMYBA2, VlMYBA1–2, VlMYBA1–3*) related to anthocyanin biosynthesis. The maximum anthocyanin levels were observed at 15 °C in light treatment as compared to a fall in anthocyanin at 35 °C in dark [206]. A positive correlation between exposure to sunlight and anthocyanin content was also studied on *Litchi Chinensis* which identified an R2R3-MYBTF encoding gene; *LCMYB1* whose expression enhanced on exposure to ABA and sunlight. The expression of this gene also correlated with tissue anthocyanin content and expression of *LCUFGT* gene [207]. The molecular switch of light signaled processes in plant is COP1 which is a negative regulator of photoreceptors and mediates TF^s^ which promote photomorphogenesis by Ub- proteasome system. Moreover, in apples higher levels of *MdMYB1* accumulate on exposure to light following which it interacts with *MdCOP1* [208]. The protein is degraded by ubiquitin dependent pathway in dark as COP1 interacts with target TF^s^ like HY5 and it regulates their breakdown and degradation via the 26S proteasome pathway [209]. In Arabidopsis, HY5 has been observed to activate *CHS (chalcone synthase)* gene and promote flavonoid build up as a response to stimulus like light and UV-B radiation. Recent reports suggest the role of UV-B induced accretion of anthocyanins through the COP1 regulated signaling in binding of HY5 to *MYB* gene promoters [210]. The exposure of UV radiation on the fruit affects the anthocyanin accumulation depending on its stage of development. The shikimate pathway genes have been reported to be upregulated by UV-A radiations in 3 weeks old grape berries as compared to UV-B and UV-C radiations which positively regulated the shikimate pathway genes by 11 weeks [211]. UV radiation has been reported to have similar upregulation of structural genes *VvANR, VvLAR1, VvLAR2* and regulatory genes *MYB5a, MYB5b, MYBPA1* in grapes [212]. In the dessert plant *Reaumuria soongorica*, the expression of flavanone-3-hydroxylase, an enzyme involved in anthocyanin biosynthesis increased under the influence of UV radiations and stress due to drought [213]. The effect of mutations on anthocyanin accumulation has been studied well in the strawberry fruit. The red color of strawberry is due to the vacuole accumulated anthocyanins. In a wild variety of strawberry (*Fragaria vesca*) with white color fruits, a mutant *RAP (reduced anthocyanin in petioles)* has been reported. This mutation was a stop codon in the synthesis of *GST* (*glutathione s-transferase)* gene. Among the eight genes in *GST* family, *RAP* is present abundantly in ripening fruits and acts as a transporter of anthocyanin. However, in cultivated strawberry, *RAP* mutant acts downstream to FvMYB10 which is the fruit specific TF^s^ and results in a reduction in fruit color [214]. Therefore, a knowledge of the impact of various environmental stimuli on TF^s^ involved in anthocyanin biosynthesis could be utilized to engineer anthocyanin production and enhance its accumulation as an essential nutraceutical in a ripened fruit.

### Structural modifications impart stability to anthocyanins

Several anthocyanins have been isolated from plant species based on a single basic flavonoid framework of carbon atoms as C6-C3-C6. The basic flavonoid structure contains one integrated aromatic ring as A ring, the B ring which is a phenyl component and the C ring comprises of a heterocyclic benzopyran [215]. The stability of color in anthocyanins is dependent on the structural modifications of the B ring. It has been reported that hydroxylation of the B ring imparts a blue hue while methylation imparts a red hue to anthocyanidins. Malvidin has been reported as the reddest anthocyanin [216]. Cyanidin, delphinidin and petunidin are more sensitive to oxidation owing to the presence of O-diphenol structure as compared to malvidin and peonidin which do not possess the hydroxyl groups at the ortho position [216]. The accumulation of anthocyanins in fruits is accompanied by an immediate modification by glycosylation, methylation, and acylation to increase their stability as vacuolar anthocyanins. In grapes, the linkage of glucose molecules to the anthocyanic structure can only be at the C3 position to form monoglycosidic anthocyanins, however, in other plants the linkage of glucose molecule can be at both C3 and C5 positions to form diglycosidic anthocyanins [217]. Though the stability of monoglucosidic anthocyanins is lesser than diglucosidic counterparts, monoglucosidic anthocyanins have more deeper color. In grapes, the principal anthocyanins reported are monoglucosides of delphinidin, peonidin, malvidin, cyanidin, petunidin and pelargonidin. Yet another structural alteration of the B ring in anthocyanins is carried out by methylation of the hydroxyl groups at the C3 or C5 positions. In grapes, divalent cation dependent OMT (O-methyl transferase) have been reported to regulate the process of methylation of flavanols and anthocyanins by preferring the 3rd and 5th position for methylation with substrates having hydroxyl groups at 3rd,4th and 5th position thus playing a vital role in anthocyanin biosynthesis [218]. Furthermore, the acylation of the sugar at the C6 position of glucose moiety with inclusion of aromatic and aliphatic functional groups can promote the chemical stability and increase structural diversity of anthocyanins. In addition to coumaric acid two stereoisomers of anthocyanidin coumaryl glucosides have been reported in grapes [219]. Some complex mechanisms like self-association, co-pigmentation, and creation of pyranoanthocyanins impart stability to the color of anthocyanins. Pyranoanthocyanins are formed during the process of fermentation or in oxygenation processes which are controlled. One of the members of this family of pyranoanthocyanins is vitisin A, which is formed as a by-product of reaction between pyruvic acid and anthocyanins (cyanidin, delphinidin, peonidin, petunidin, malvidin) bearing either glycosyl, acetyl glycosyl or coumaroyl glycosyl groups and it has been reported to play an important role as an intermediate in alcohol fermentation. Another example of pyranoanthocyanins is vitisin B, reported as the primary product of ethanol oxidation and is formed by a chemical reaction between acetaldehyde and malvidin possessing either glycosyl, acetyl glycosyl or coumaroyl glycosyl groups [220].

### Transport and storage of anthocyanins

Anthocyanin synthesis takes place at endoplasmic reticulum following which they are transported to the anthocyanic vacuolar inclusions for storage. Electron microscopy of plant cells exhibiting anthocyanin pigments depict anthocyanic vacuolar inclusions (AVI) which are formed because of hydrogen bonding of anthocyanin to the protein matrix. The concentration of anthocyanins have been reported to be intensified in areas rich in AVI [221]. These vacuoles are formed by acylated anthocyanins and glycosylation of the acylated anthocyanins reduces their tendency to form AVI [222]. There are many regulatory systems which mediate anthocyanin transport in plants like MATE (multidrug and toxic extrusion), GSTs, allergen Fra a 1 and ABC (ATP-binding cassette) proteins [223, 224]. Among the other transporters, GSTs play a crucial role, as a decline in their activity results in visual pigment loss resulting in phenotypes like bz2 (Bronze-2) in maize, an9 (anthocyanin 9) in petunia and tt19 (transparent testa 19) in Arabidopsis [225]. Several studies have reported the role of tt19 protein as a carrier for seclusion and transport of anthocyanins into the vacuole from the cytosol [226]. GST’s have also been identified in fruit and flower pigmentation like LcGST4 in lychee [227] Riant in peach [228] and MdGST in apple [229].

### Mutual exclusion of pigments with anthocyanins

Anthocyanins exhibit a property of co-pigmentation which leads to stabilization of color in plants. The co-pigments can be of a wide range from alkaloids, other flavonoids, nucleotides, organic acids, and metals. The complexes thus formed result in increased absorption intensity and change in wavelength leading to increased hue intensity of anthocyanins [230]. Many factors influence the magnitude of co-pigmentation like structure, concentration of the anthocyanin and their co-pigment, the solidity of ionic bonding and their molar ratio, temperature and pH [231]. While carotenoids and anthocyanins coexist, a mutual exclusion has been reported for betalains and anthocyanins. An interesting observation is the absence of anthocyanin from any betalain cumulating family. It has been reported that the plants accumulating betalains do express a few flavonoid biosynthetic enzymes and accumulate flavanols, however the final step regulated by ANS of the flavonoid pathway is not carried out due to the presence of truncated ANS enzyme in plants which cannot cumulate betalain [232].

## Conclusion

Considering the importance of pigments in nutraceuticals and varied industries and their accumulation across different developmental stages of ripening, decisions regarding the optimum time for harvest could be made based on the pigments desired. Harvesting of un-ripened fruits could be a potential source of nutraceuticals like chlorophylls’ and xanthophylls’, the breaker stage would contribute to a supply of both chlorophyll and carotenoids, while fully ripened fruits will be a reservoir of pigments like anthocyanins and carotenoids along with their cleavage products, many of which have important clinical functions [39, 44, 158, 159, 233–235]. Many non-invasive techniques could be used to determine the type and quantity of pigments at different stages of ripening [236] ranging from the age-old colorimeters [237] to the more recent electronic nose technique [238]. The other commonly used light transmittance techniques are visible imaging [239], visible and infrared spectroscopy (VNIR) spectroscopy [240], multispectral and fluorescence imaging [241], CT and MRI scan [242]. Utilizing the visual signals of fruit ripening in terms of color, indicative of pigment accumulation, the desired pigments could be harvested and processed at an individual level based on its bioactivity. Recent evidence indicates that the regulatory mechanism involved in expression of ripening related genes are more complex than imagined earlier. To further fine tune the expression of pigments in fruits, post-transcriptional mechanisms along with RNA splicing would play the key role and offer novel substrate for the upsurge of genetic variables and grant an evolutionary flexibility to the expression of fruit pigments [9].

Also, extensive research on pigment biosynthetic pathways, stability, degradation, storage, and the underlying regulatory mechanisms highlights ways to engineer pigment content with the aid of biotechnological advances and genome editing [243]. Denovo domestication via molecular breeding using CRISPR/Cas9 genome engineering strategy has promoted an increase in pigment content such as lycopene by 500% in engineered lines in comparison to wild tomato varieties [244]. Manipulating the biosynthesis and stockage of secondary metabolites also adds the potential of improving the nutritional and health benefits, flavors and aromas of fruits and vegetables that has the potentially to encourage a more diverse and healthy diet [44]. Plant secondary metabolites and their breakdown products have a high degree of pharmaceutical potential, which is still largely unexplored. Many have been reported to have anti-cancer and anti-inflammatory properties and can be used to treat mental health. Saffron, for example, (30 mg/day^− 1^) is used to treat mild to moderate depression with no side effects [245]. These data only strengths the view that increasing the content of these clinically relevant compounds in foods could potentially have a wide impact on human health.

Some of these compounds have also been used as biopesticides and bioherbicides making them a potential source of alternate farming compounds that could be used to reduce our needs on chemicals that may be harmful to the environment, wildlife, and insect populations. Increasing their content and marketable yield either through breeding, selective harvesting or genetic engineering could reduce the overall costs, making them more attractive alternatives to current use chemicals.

Another opportunity to potentially manipulate fruit metabolite content is to manipulate primary metabolism [246, 247] directly in the fruit. Several authors have suggested that fruit carry out carbon capture, either directly through stomata on their surface, or through the recycling of respiratory carbon via the Calvin-Benson cycle [10, 78–81].

This review generates new hypothesis for future research as different stages of ripening induce several structural changes in pigments resulting in the formulation of novel unexplored pigments, which may prove to be a boost to the existing era of green technology.

## Data Availability

Not Applicable.
